# Proteomic aptamer analysis reveals serum markers that characterize preclinical systemic sclerosis (SSc) patients at risk for progression toward definite SSc

**DOI:** 10.1186/s13075-023-02989-w

**Published:** 2023-01-27

**Authors:** Chiara Bellocchi, Shervin Assassi, Marka Lyons, Maurizio Marchini, Chandra Mohan, Alessandro Santaniello, Lorenzo Beretta

**Affiliations:** 1 Department of Clinical Sciences and Community Health, University of Milan, Milan, Italy; 2 Scleroderma Unit, Referral Center for Systemic Autoimmune Diseases, Fondazione IRCCS Ca’ Granda, Ospedale Maggiore Policlinico, Milan, Italy; 3 Department of Internal Medicine – Rheumatology, University of Texas Health Science Center at Houston, Houston, TX USA; 4 Department of Biomedical Engineering, University of Houston, Houston, TX USA

**Keywords:** Systemic sclerosis, Proteomic, Preclinical stage, Disease progression, Vasculopathy

## Abstract

**Background:**

The study of molecular mechanisms characterizing disease progression may be relevant to get insights into systemic sclerosis (SSc) pathogenesis and to intercept patients at very early stage. We aimed at investigating the proteomic profile of preclinical systemic sclerosis (PreSSc) via a discovery/validation two-step approach.

**Methods:**

SOMAcan aptamer-based analysis was performed on a serum sample of 13 PreSSc (discovery cohort) according to 2001 LeRoy and Medsger criteria (characterized solely by Raynaud phenomenon plus a positive nailfold capillaroscopy and SSc-specific antibodies without any other sign of definite disease) and 8 healthy controls (HCs) age, gender, and ethnicity matched. Prospective data were available up to 4±0.6 years to determine the progression to definite SSc according to the EULAR/ACR 2013 classification criteria. In proteins with relative fluorescence units (RFU) > |1.5|-fold vs HCs values, univariate analysis was conducted via bootstrap aggregating models to determine the predicting accuracy (progression vs non-progression) of categorized baseline protein values. Gene Ontologies (GO terms) and Reactome terms of significant proteins at the adjusted 0.05 threshold were explored. Significant proteins from the discovery cohort were finally validated via ELISAs in an independent validation cohort of 50 PreSSc with clinical prospective data up to 5 years. Time-to-event analysis for interval-censored data was used to evaluate disease progression.

**Results:**

In the discovery cohort, 286 out of 1306 proteins analyzed via SomaScan, were differentially expressed versus HCs. Ten proteins were significantly associated with disease progression; analysis through GO and Reactome showed differentially enriched pathways involving angiogenesis, endothelial cell chemotaxis, and endothelial cell chemotaxis to fibroblast growth factor (FGF). In the validation cohort, endostatin (HR=10.23, CI95=2.2–47.59, *p*=0.003) was strongly associated with disease progression, as well as bFGF (HR=0.84, CI95=0.709-0.996, *p*=0.045) and PAF-AHβ (HR=0.372, CI95=0.171–0.809, *p*=0.013)

**Conclusions:**

A distinct protein profile characterized PreSSc from HCs and proteins associated with hypoxia, vasculopathy, and fibrosis regulation are linked with the progression from preclinical to definite SSc. These proteins, in particular endostatin, can be regarded both as markers of severity and molecules with pathogenetic significance as well as therapeutic targets.

**Supplementary Information:**

The online version contains supplementary material available at 10.1186/s13075-023-02989-w.

## Background

Systemic sclerosis (SSc) is a rare disease mainly characterized by vasculopathy, immune-system activation, and fibrosis that can potentially affect any organ. Patients can present different clinical manifestations along with different clinical subsets of disease. The pathogenesis underlying this variety of disease features is complex and still incompletely unrevealed [1]. A limited number of subjects can be intercepted and identified at a preclinical stage of disease presenting solely Raynaud phenomenon (RP), SSc-specific autoantibodies, and/or typical abnormalities at a nailfold video-capillaroscopy (“scleroderma pattern”) without any other clinical fibrotic features [2, 3]. Patients with preclinical SSc (PreSSc) have a risk of disease progression into a definite SSc of about 50% within 5 years of diagnosis [4, 5]. The biological characterization of this group of patients is highly relevant for gaining insight into SSc pathogenesis and mechanisms of disease progression.

Proteins are in closer proximity to pathological processes and therefore are functionally more relevant than information obtained from DNA- or RNA-level studies. Several studies have explored the circulating levels of cytokines, chemokines, and other molecules in SSc subsets. Limited by technologies available, the majority of studies focused only on a small group of proteins chosen a priori, while larger panels of proteins were just analyzed in a few studies [6–8]. Cytokines and chemokines related to endothelial dysfunctions, fibrosis, and adhesion molecules clearly emerged as markers of disease severity and stage [9–11]. Moreover, the serum proteomic profile of SSc patients is clearly different from that of healthy controls also correlating more closely with molecular dysregulations of affected organs such as skin [6, 8].

In the present project, with a two-step strategy, we aimed at exploring the proteomic profile of a well-characterized group of PreSSc compared with matched healthy controls, using a validated innovative and comprehensive platform based on a library of aptamers. We then performed a validation analysis in a longitudinal cohort of 50 PreSSc through an enzyme-linked immunosorbent assay (ELISA) to confirm the circulating factors that are robustly associated with disease progression.

## Methods

### Patients and controls

Two different cohorts of patients were examined pursuing a 2-step approach with discovery and validation strategy.

As a first step, 13 PreSSc, defined according to LeRoy and Medsger criteria (Raynaud phenomenon plus a positive nailfold capillaroscopy and SSc-specific auto-antibodies without any other sign of definite disease) [2] with available baseline and subsequent clinical data at approximately 4 years as well as baseline aliquoted serum samples, were included in the discovery cohort. Clinical data, including the occurrence of puffy fingers, sclerodactyly, telangiectasia, lung fibrosis, pulmonary arterial hypertension, scleroderma renal crisis alone or in combination indicative of the progression to definite SSc (thus with a minimum score of 9) according to the EULAR/ACR 2013 classification criteria [12, 13] were retrieved from medical records allowing the evaluation of disease progression as previously described [10]. Patients with a definite SSc but with puffy fingers without skin fibrosis were considered as limited cutaneous SSc (lcSSc). At baseline serum samples were collected and stored at −80 °C; samples from 8 ethnically-, age- and sex-matched healthy controls (HCs) were collected as well. Cases and controls from the discovery cohort were screened via the SomaScan® aptamer analysis to find proteins of interest linked to progression from PreSSc to definite SSc patients.

As a second step, 50 independent PreSSc patients with baseline serum samples and available prospective clinical data at 5 years, were considered as the validation cohort; serum from the validation cohort was aliquoted at a different time from those of the discovery cohort. ELISA (RayBiotech Life, Inc. MyBioSource Cloud-Clone Corp) of serum samples was used to validate the relevant proteins found in the discovery cohort.

The study was performed in accordance with the Declaration of Helsinki and approved by the local ethic committee (approval n. 559_2018) and patients signed informed consent to participate in the study.

### Aptamer analysis

Comprehensive targeted proteomics was performed using the SomaScan® assay as described [14] interrogating the levels of 1306 different proteins. All samples were clarified by centrifugation before use and were screened using the SomaScan® aptamer-based screening platform at the Houston Omics Collaborative (https://hoc.bme.uh.edu/). This assay uses aptamer–protein interactions to detect proteins within a sample. In the assay, aptamer-coated streptavidin beads are first added to the sample to allow the aptamers to bind to the proteins. Next, the bound proteins are biotinylated, and the aptamer–protein complexes are cleaved from the streptavidin beads. These aptamer–protein complexes are then conjugated to a second streptavidin bead, and aptamers are separated from the proteins. The aptamers are then collected from the sample and quantitated by hybridization to a DNA microarray. The final output is the relative fluorescence unit (RFU) for each protein; these RFU values were then normalized and statistically analyzed. The limit of detection (LOD) of the aptamer-based scan was determined by spiking proteins into buffer before the assay. The limits of quantitation (LOQ) were established along with the LOD, and the median lower LOQ value is approximately 3-fold higher than the LOD.

### Statistical analysis

#### Predictive accuracy in the discovery cohort

Due to the limited sample size, only proteins whose RFUs were increased or decreased compared to HCs were considered. To this end, a log fold-change (FC) ≥ 0.585 was used as cut-off. Considering 13 cases (Nc = 13) and 8 controls (Nhc = 8), the FC was calculated from individual RFU values.

Bootstrap aggregating (bagging with 100 resamplings) was used to determine the accuracy of categorized baseline protein values in predicting the subsequent status (progression vs non-progression) at the last available observation. In each in-bag sample, the threshold to define the risk of evolution was considered the median value RFU of each protein, whose predictive accuracy was calculated from 2 × 2 tables in the corresponding out-of-bag samples. A 10,000-fold step-down permutation approach (Tmax method) was then used to assess the significance of predictions and to correct for family-wise error rates [15], a nominal 0.05 value was then used. A custom-code written in python by LB on top of the Scikit-learn machine learning libraries [16] was used for the analyses.

#### Gene ontology (GO) analysis

Enrichment analysis of significant aptamers found in the discovery phase was performed using the ShiniGO web application [17, 18]. To this end, the corresponding genes were used to find significant GOs at the biological process level and to explore Reactome pathways.

#### Survival analysis in the validation set

To better assess the prognostic implications of individual proteins identified in the discovery set, and to exploit all the available information, prospective data from the validation cohort were used. Time-to-progression was explored using the Cox-regression method for interval-censored data after Box-Cox transformation of data to ensure normality [19]. Significant analytes were categorized after cutpoint estimation on right-censored samples according to the method described by Contal and O’Quigley [20]; the Turnbull method for interval-censored data [21] was used to calculate survival estimates of dicothomized proteins and the corresponding P values were calculated with the generalized logrank test for right-censored failure time data according to Sun [22]. Missing data were first imputed according to Beretta and Santaniello [23] using the rkNN-imputer Scikit-learn library [17] setting the number of neighbors equal to 3. Time-to-event analyses were done using R 4.0.5 [R Core Team, 2021] with the AID [24], icenReg [25], the interval [26], and the survMisc: Miscellaneous Functions for Survival Data [v0.5.5] [27] packages.

Throughout the article, descriptive statistics are presented as mean ± standard deviation except for skewed values that are presented as median and interquartile ranges.

## Results

### Ten proteins in the discovery cohort associated with fibrogenesis and angiogenesis are associated with progression to definite SSc

The 13 preclinical-SSc subjects included in the discovery cohort aged 53.5 ± 6.3 years, were mostly females (*n*=10, 76.9%), and all tested positive for antinuclear antibodies (ANA); anticentromere antibodies (ACA) were found in 8 subjects (61.5%), anti-topoisomerase I antibodies (ATA) in 3 (23.1%) and ANA with nucleolar staining in 2 (15.4%). After 4 years, 7 patients (54%) progressed into definite SSc, with lcSSc skin features (6 presenting solely puffy fingers). Non-progressors and progressors were similar regarding overall observation time and baseline characteristics: ACA positivity, 66% vs 57%; forced vital capacity (FVC) % of predicted values, 101.5 ± 12.4 vs 112.7 ± 5.8 diffusing capacity for carbon monoxide (DLco), 95.5 ± 20.3 vs 90.3 ± 11 (Supplemental Table 1).

Two hundred eighty-six proteins out of the 1306 analyzed via the SomaScan® assay were differentially expressed in comparison with 8 matched healthy controls (females, *n*=7, 87.5%; age 55.8 ± 4.1 years) and were further considered for the analysis. Bagging experiments after resampling with permutation, showed that ten proteins were significantly associated with the development of definite SSc in preclinical samples: NKp30, Endostatin, basic fibroblast growth factor (bFGF), extracellular matrix protein 1 (ECM1), FGF18, phospohexose isomerase (PHI), Fibronectin 1.3 (FN1.3), Ubiquitin +1, platelet-activating factor acetylhydrolase-β subunit (PAF-AHβ), fatty acid binding protein (FABP) (Table 1).

**Table 1:** Proteins of interest in the discovery cohort after bagging validation

Target	Gene	Accuracy^a^	P*
NKp30	NCR3	0.835	0.0071
Endostatin	COL18A1	0.835	0.0075
bFGF	FGF2	0.83	0.0075
ECM1	ECM1	0.766	0.0248
FGF 18	FGF18	0.755	0.0324
PHI	GPI	0.749	0.0345
Fibronectin 1.3 (FN1.3)	FN1	0.740	0.0402
Ubiquitin +1	RPS27A	0.740	0.0402
PAF AHβ subunit (PABF)	PAFAH1B2	0.734	0.0459
FABP	FABP1	0.732	0.0467

*Family-wise corrected *p* values of bagging accuracy after 10,000 permutations

^a^Average accuracy to predict evolution after 100 resampling with replacement (bagging accuracy)

**Fig. 1:**
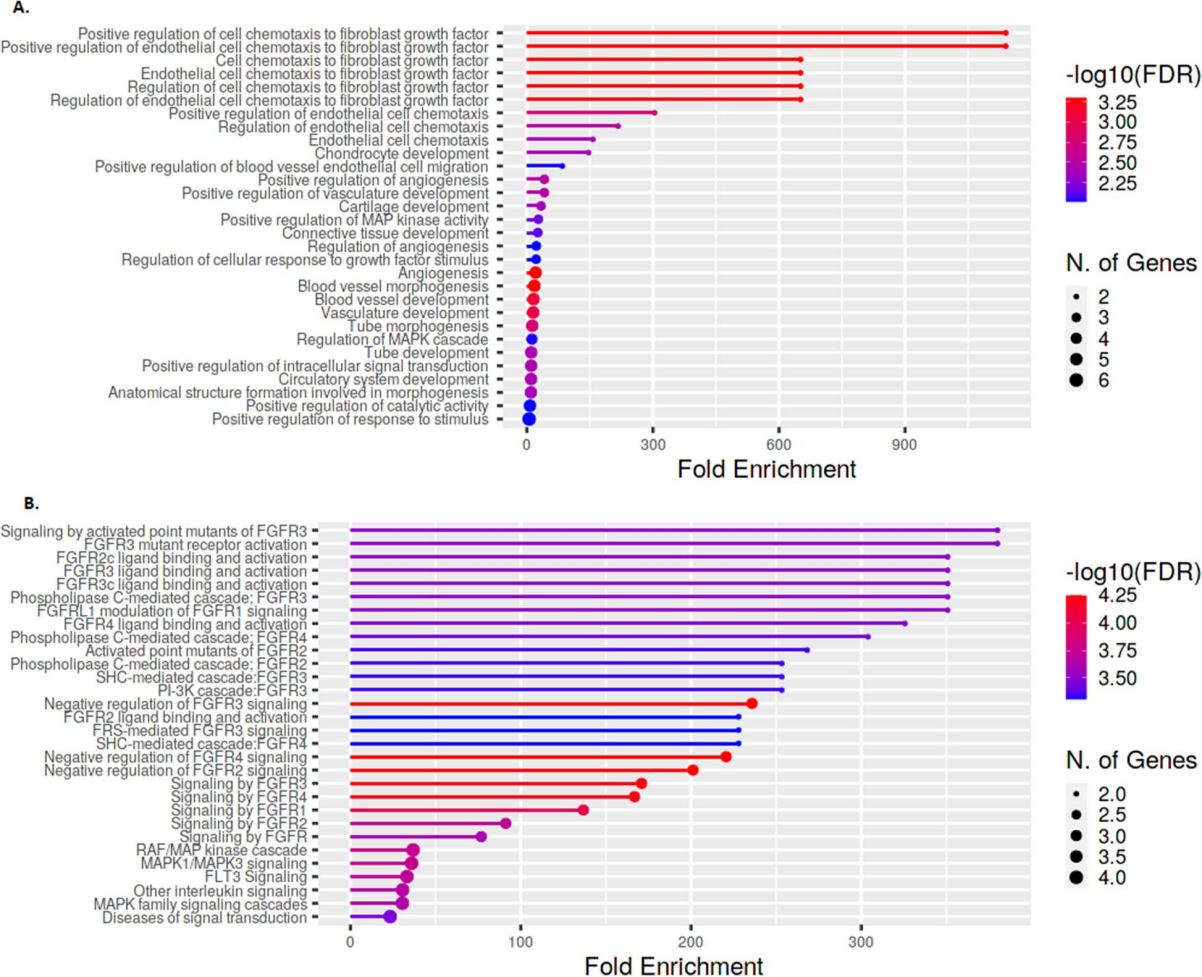
Enrichment analysis in the discovery set. Enrichment analysis: genes related to the 10 proteins selected in the discovery cohort (see Table 1). **A** Gene Ontologies (GO) at the biological process level. **B** Reactome pathways

Analysis of GO biological processes showed a few differentially enriched pathways involving angiogenesis, endothelial cell chemotaxis, and endothelial cell chemotaxis to fibroblast growth factor (Fig. 1A). These processes are mostly mediated by binding and activation of FGF receptors (FGFR) as indicated by Reactome enrichment analysis (Fig. 1B).

### Validation of proteins with prognostic significance in survival models

The characteristics of the 50 patients included the validation cohort are reported in Table 2.

**Table 2:** Demographic and clinical characteristics of the validation cohort

Feature	Preclinical SSc (***n*** = 50)	Non-progressors (***n*** = 30)	Progressors (***n*** = 20)
Age, years	55.9 ± 14.04	56.8 ± 13.18	54.55 ± 15.15
Females, *n* (%)	44 (88)	26 (86.7)	18 (90)
Ethnicity Caucasian, *n* (%)	50 (100)	30 (100)	20 (100)
ANA, n (%)	47 (94)	28 (93)	19 (95)
ACA, n (%)	32 (64)	17 (57)	15 (75)
Anti-topoisomerase I, *n* (%)	8 (16%)	3 (1)	5 (25)
Other autoantibodies, *n* (%)	20 (40)	11 (36.67)	9 (45)
RP duration, months	128.4 ± 115.7	154.3 ± 120.8	90 ± 95.41
FVC, % predicted	115.6 ± 16.04	116.7 ± 17.8	112.5 ± 12.8
DLCO, % predicted	84.2 ± 17.2	86.9 ± 17	80.1 ± 16.7
Evolution, *n* (%)	20 (40)	0 (0)	20 (100)
Skin progression, *n* (%)	16 (32)	0	16 (80)
GERD, *n* (%)	19 (38)	9 (30)	12 (60)
Telangiectasia, *n* (%)	6 (12)	0	6 (30)
Low-dose aspirin, *n* (%)	42 (84)	26 (87)	16 (80)
CCB, *n* (%)	36 (72)	21 (70)	15 (75)

Clinical features of 50 preclinical systemic sclerosis (SSc) patients included in the validation cohort and in relation to the progression/non-progression to definite SSc within 5 years from blood draw. *ANA* Antinuclear antibodies, *ACA* Anticentromere antibodies, *RP* Raynaud’s phenomenon, *FVC* Forced vital capacity, *DLCO* Diffusing capacity for carbon monoxide, *GERD* Gastroesophageal reflux disease, *CCB* Calcium-channel blockers

**Fig. 2:**
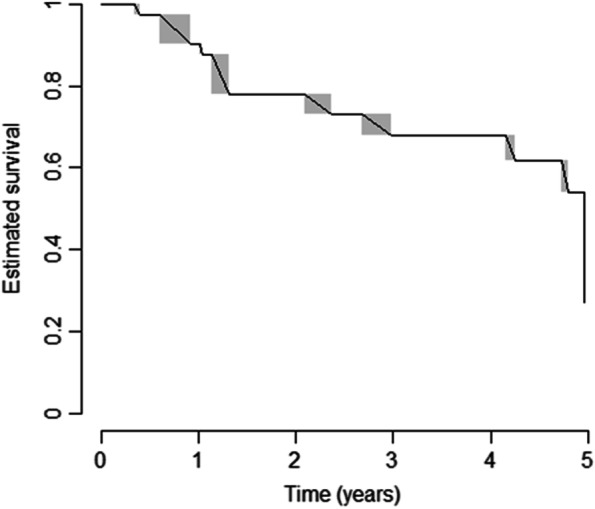
Estimated time-to-evolution in the validation cohort. Survival estimates as calculated by the Turnbull’s method, in the validation cohort; T0 = blood draw

Twenty subjects (40%) did progress into a definite SSc at the end of the 5-year observation period; the overall estimated 5-year time-to-evolution in the validation cohort is represented in Fig. 2. The prototypical sign of progression was skin involvement, namely puffy fingers in 13 cases (65%) and overt skin fibrosis in 7 with limited cutaneous SSc (lcSSc) (35%); telangiectasia did appear in combination with the above in 6 cases (30%).

Patients with Raynaud’s duration shorter than 10 years (*p* = 0.0425) at baseline or with reflux disease (*p* = 0.014) had shorter times to progression while none of the other baseline clinical characteristics was associated with time-to-progression (Supplemental Table 2 and Supplemental Figs. 1 and 2).

**Fig. 3:**
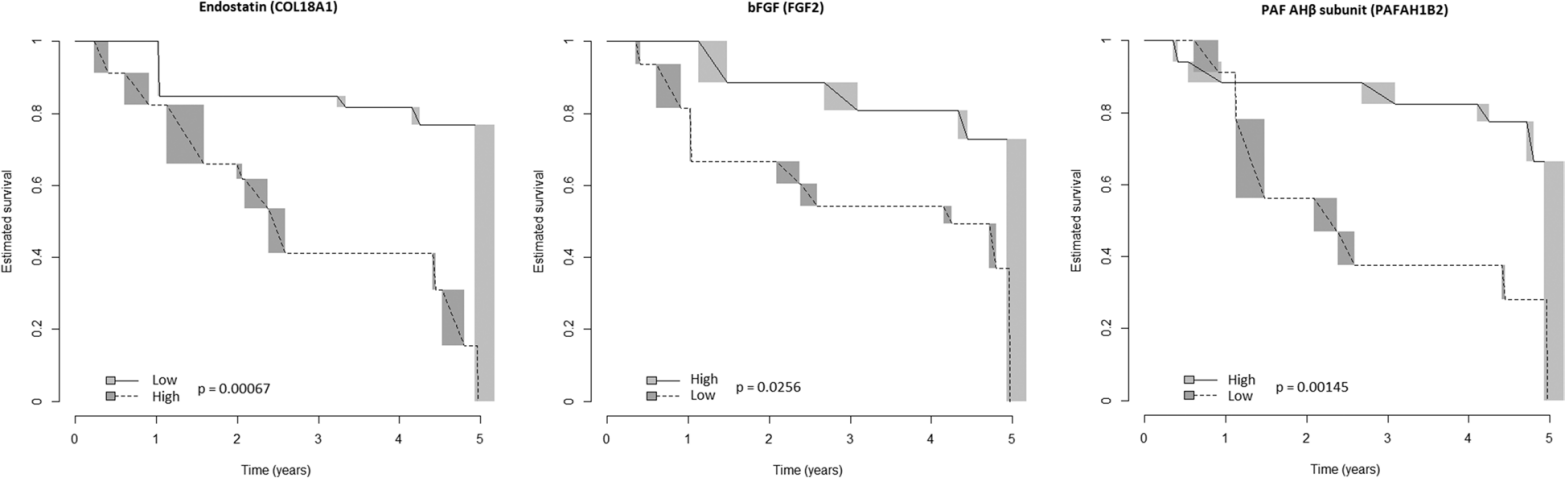
Risk of evolution to definite systemic sclerosis in the validation cohort (replicated proteins). Survival estimates (Turnbull’s method) for dicothomized proteins (high/low serum levels) with prognostic significance in the validation cohort; time, years from blood draw. bFGF, basic fibroblast growth factor; PAF-AHβ, platelet-activating factor acetylhydrolase subunit beta

**Fig. 4:**
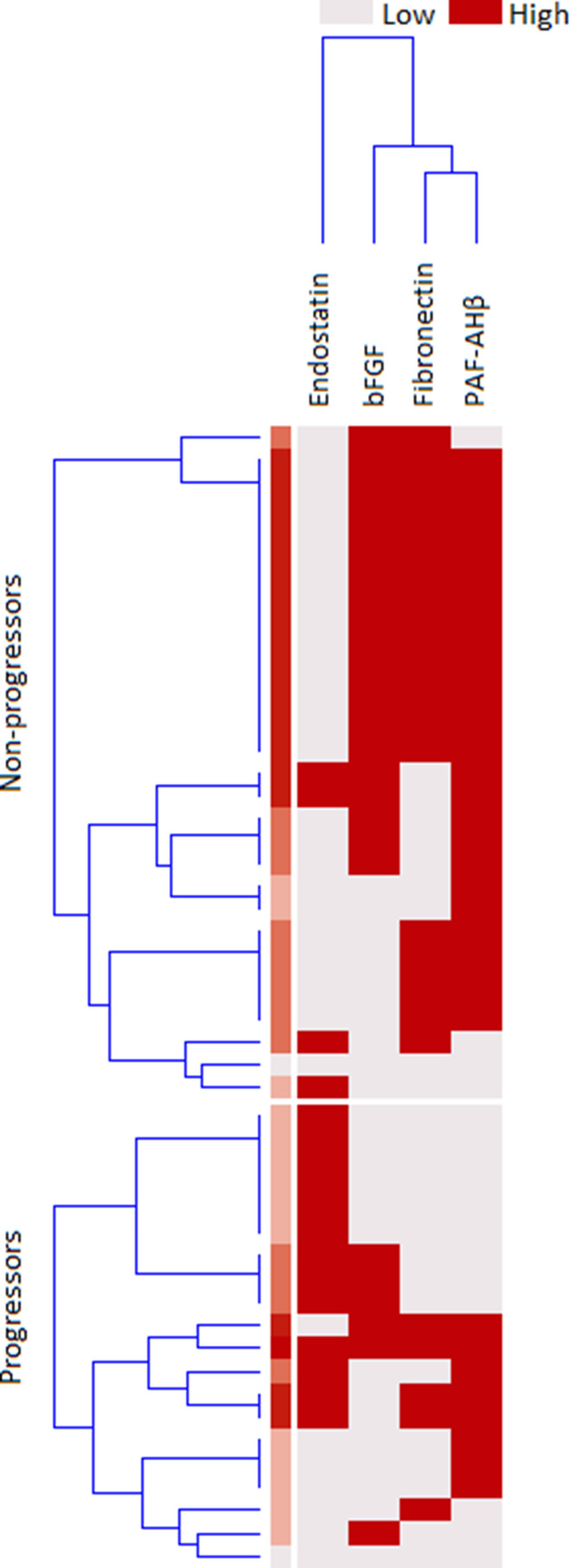
Distribution of categorized proteins in the validation cohort. Distribution of cases with high or low validated protein levels in preclinical systemic sclerosis patients who did evolve (progressors) or who did not (non-progressors) into definite systemic sclerosis. Dicothomization was performed as described in the main text; for endostatin risk is associated with high serum levels; for the basic fibroblast growth factor (FGF) and platelet-activating factor acetylhydrolase subunit beta (PAFAH1B2) risk is associated with low serum levels; clusterization made on the basis of serum concentrations

ELISA confirmation assay provided technically valuable results for all the 10 analytes selected after step 1, but Ubiquitin +1; hence, step 2 analysis was restricted to 9 molecules. Of these, Endostatin (hazard ratio for transformed data [HR] = 10.23, CI_95_ = 2.2–47.59, *p* = 0.003), bFGF (HR = 0.84, CI_95_ = 0.709–0.996, *p* = 0.045) and PAF-AHβ (HR = 0.372, CI_95_ = 0.171–0.809, *p* = 0.013) were significantly associated with progression after Cox-regression analysis for interval-censored data. Cut-point estimation showed that proteins were found to be associated with a reduced time-to-evolution: endostatin (cut-off ≥ 124 pg/mL, *p* = 6.65 × 10^−4^), bFGF (cut-off < 4.9 pg/mL, *p* = 0.02563), and PAF-AHβ (cut-off < 2.1 ng/mL, *p* = 0.00145) (Fig. 3). The rough distribution of dicothomized proteins according to the abovementioned thresholds, regardless of the time-to-evolution, in progressors and non-progressors is shown in Fig. 4.

## Discussion

The study of SSc in its preclinical phase is highly relevant to understand the pathophysiological alterations that sustain the development into a clinically evident fibrotic disease and, consequently, to discover potential avenues of early intervention. Nonetheless, this endeavor has seldom been undertaken and mostly in cross-sectional studies [10, 13, 28–33].

The main finding of ours is that 3 proteins with angiogenetic and fibrotic processes regulation significance are differentially expressed in preclinical SSc patients according to the future 5-year progression, and namely endostatin, bFGF, and PAF-AHβ. These findings support the notion that vasculopathy is fundamental in the development of SSc and of its fibrotic manifestations [34] and that microvascular damage is strictly related with the progression of scleroderma [35]. In details, it seems that patients with altered markers of angiogenesis are at risk of progression as compared to subjects with a more indolent circulatory disease, as also testified by the fact that a longer history of RP duration in absence of pivotal signs of definite SSc is also associated with lower rates of progression (Supplemental Fig. 1). Additionally, we showed that the presence of gastro-esophageal reflux was significantly associated with a shorter time of progression, in line with previous findings indicating that in PreSSc patients non-circulatory clinical signs are associated with an increased risk of progression [5, 36].

Among the proteins associated with disease progression, endostatin emerged as the one most strongly related with the passage from preclinical to definite SSc (Fig. 3). Increased serum levels of endostatin have already been described in SSc [37, 38] also correlating with the severity of vascular manifestations [37]. Endostatin is an endogenous inhibitor of proliferation and migration of endothelial cells and angiogenesis [39] that is upregulated in kidney and cardiovascular diseases [40] as well as in patients with peripheral vascular disease [41]. Endostatin is released during ischemia-reperfusion and hypoxia [42–44] and hence it may be postulated that in preclinical SSc this molecule is a marker of a more severe form of vasculopathy, as well as an anti-angiogenic factor that promotes disease progression. Endostatin has also anti-fibrogenic functions [45] and its serum increase may also reflect a feedback loop in the attempt to control and reduce upcoming fibrosis and to regulate the collagen turnover.

bFGF is a molecule with pleiotropic effects, mainly promoting angiogenesis, and fibroblast proliferation and that regulates fibrotic processes preventing fibrosis deposition through the inhibition of TGF-β mediated collagen deposition [46]. Its function is upstream of other specialized growth factors, such as vascular endothelial growth factor (VEGF) [47], whose function is tightly regulated by endostatin [48]. bFGF usually increases in response to hypoxic stimuli to promote neoangiogenesis [49] and bFGF levels were found to be mostly undetectably low in patients with SSc [50]. Low bFGF levels in preclinical SSc at risk for progression would mirror a condition of increased vasculopathy and defective response to the hypoxic condition [49, 51, 52]. Conversely, it may be argued that high bFGF levels under hypoxic conditions would act as a protective angiogenetic mechanisms in patients with slow progression rates regulating at the same time collagen deposition.

Shorter times to progression were also observed in patients with low PAF-AHβ serum levels. PAF-AH degrades the platelet-activating factor [53] counteracting its main effect, including leukocyte chemotaxis, adhesion and degranulation, endothelial permeability and dysfunction, vasoconstriction, and the promotion of the release of proinflammatory cytokines, including interleukin (IL)-1 and IL-6 [54]. Notably, PAF-AH may prevent ischemia-reperfusion [55] and is down-regulated in hypoxic rat models [56]. These observations suggest that high levels of PAF-AHβ may be protective against the progression of endothelial dysfunction in scleroderma as well as be a marker of a milder form of vasculopathy that is at lower risk of evolution.

Taken together, our results suggest that soluble factors associated with hypoxia and vasculopathy, are linked with the transition from preclinical to definite SSc and that these may be regarded both as markers of severity and molecules with pathogenetic significance. A quantification of SSc-related vasculopathy, as for instance nailfold video-capillaroscopy (NVC) scores, would have helped to better establish a correlation between endothelial damage, vasculopathy, and circulating markers, yet NVC data were not available in our patients. Nonetheless, NVC scleroderma patterns were found to be predictive of clinical complications of the disease [57, 58] and preclinical patients with severe NVC had shorter times to definite SSc compared with those with less severe patterns [59], indirectly supporting our findings.

The discovery-validation strategy we applied guarantees that our results are reproducible and strongly mitigates the risk of false-positive findings. Nonetheless, we are aware that other potential candidates may have been overlooked because of the selection procedure we used in the discovery phase. Because of the low sample size, we decided to restrict the analysis to a panel of candidates that were differentially expressed as compared to healthy controls. Moreover, statistical results were adjusted for multiple tests and albeit permutations correct type I errors less conservatively than other methods (i.e., Bonferroni or false-discovery rate) [60] the risk of type II errors (e.g. loss of power) is still substantial.

Our cohort was composed uniquely by Caucasian subjects, therefore the generalization of our results to other ethnicities should be assessed by future studies.

Another shortcoming of our study is related to the relatively over-representation of patients with ACAs antibodies and to the long-lasting RP duration. These characteristics clearly underlie the difficulty of intercepting patients with more aggressive disease in favor of subjects that will eventually develop a limited cutaneous form of SSc, as also observed in a multicenter study of very early SSc subjects [5]. This almost unavoidable selection bias clearly warns that caution should be exercised in applying our findings to all PreSSc patients, even if our findings are biologically plausible.

## Conclusions

In summary, PreSSc showed a distinct protein profile and proteins that are related to hypoxia, vasculopathy, and collagen turnover, which emerged at characterizing the progression from a preclinical stage of SSc to a definite one. In particular, endostatin was the protein most strongly associated with disease progression, and it is worthwhile to further investigate its mechanistic roles for its possible pathogenetic role in SSc development and its therapeutic potential.

## Supplementary Information

**Additional file 1: Supplemental Table 1.** Clinical characteristics of patients in the discovery (step 1) cohort. **Supplemental Table 2.** Clinical variables associated with disease progression in the validation cohort. **Supplemental Figure 1.** Survival estimates in relation to the duration of Raynaund’s phenomenon at baseline in the validation cohort. **Supplemental Figure 2.** Survival estimates in relation to the presence of reflux disease at baseline in the validation cohort.

## Data Availability

The datasets used and/or analyzed during the current study are available from the corresponding author on reasonable request.

## Abbreviations

SSc: Systemic sclerosis
PreSSc: Preclinical systemic sclerosis
HCs: Healthy controls
RFU: Relative fluorescence units
GO: Gene Ontology
FGF: Fibroblast growth factor
ELISA: Enzyme-linked immunosorbent assay
LOD: Limit of detection
LOQ: Limits of quantitation
ANA: Antinuclear antibodies
ACA: Anticentromere antibodies
ATA: Anti-topoisomerase I antibodies
FVC: Forced vital capacity
DLco: Diffusing capacity for carbon monoxide
bFGF: Basic fibroblast growth factor
ECM1: Extracellular matrix protein 1
PHI: Phospohexose isomerase
FN1.3: Fibronectin 1.3
PAF-AHβ: Platelet-activating factor acetylhydrolase-β subunit
FABP: Fatty acid binding protein
FGFR: FGF receptors
VEGF: Vascular endothelial growth factor
IL: Interleukin
NVC: Nailfold video-capillaroscopy
