# Supplementary material for: Proteomic aptamer analysis reveals serum markers that characterize preclinical systemic sclerosis (SSc) patients at risk for progression toward definite SSc
Source: Arthritis Res Ther. 2023 Jan 27;25:15. doi: 10.1186/s13075-023-02989-w (PMC9881382; doi:10.1186/s13075-023-02989-w)

**Supplemental Table 1 – Clinical characteristics of patients in the discovery (step 1) cohort**

| **Features** | **PreSSc (13)** | **Non-Progressors (6)** | **Progressors (7)** | **HC (8)** |
| --- | --- | --- | --- | --- |
| **Age, mean years ± SD** | 53.5 ± 6.3 | 52 ± 4.7 | 54.3 ± 7.6 | 55.8 ± 4.1 |
| **Gender, Female n (%)** | 11 (84.6) | 5 (83.3) | 6 (85.7) | 7 (87.5) |
| **Time Ryanaud** | 8.1 (5.6) | 8.8 (5.4) | 7.5 (6.2) | N.A. |
| **Ethnicity, n (%)** |  |  |  |  |
| **Caucasian** | 13 (100) | 6 (100) | 7 (100) | 8 (100) |
| **Autoantibodies n (%)** |  |  |  |  |
| **ANA** | 13 (100) | 6 (100) | 7 (100) | N.A. |
| **ACA** | 8 (61.5) | 4 (66.7) | 4 (57.1) | N.A. |
| **ANA nu** | 2 (15.4) | 1 (16.7) | 1 (14.3) | N.A. |
| **Anti-Scl70** | 3 (23.1) | 1 (16.7) | 2 (28.6) | N.A. |
| **Time of observation yrs ± SD** | 4 ± 0.6 | 3.8 ± 0.4 | 4.1 ± 0.7 | N.A. |
| **FVC% mean ± SD** | 107.5 ± 10.7 | 101.5 ± 12.4 | 112.7 ± 5.8 | N.A. |
| **DLCO% mean ± SD** | 92.7 ± 15.5 | 95.5 ± 20.3 | 90.3 ± 11 | N.A. |
| **Skin fibrosis** | 0 (0) | 0 (0) | 7 (100) | N.A. |

**Supplemental Table 2 – Clinical variables associated with disease progression in the validation cohort**

| **Variable** | **P value** |
| --- | --- |
| RP > 120 months | 0.0425 |
| GORD | 0.0141 |
| ACA | 0.2368 |
| Anti-Topoisomerase I | 0.1332 |
| Age ≥ 55 years | 0.4531 |
| Male gender | 0.9635 |
| DLco ≤ 80% of predicted | 0.5826 |
| Use of aspirin | 0.734 |
| Use of CCB | 0.4769 |

RP, Raynaud’s phenomenon; GORD, gastroesophageal reflux diseases; ACA, anticentromere antibodies; DLco, diffusing capacity for carbon monoxide; CCB, calcium channel blockers; P, univariate p value after generalized logrank test for right-censored failure time data.

**Supplemental Figure 1 – Survival estimates in relation to the duration of Raynaund’s phenomenon at baseline in the validation cohort**


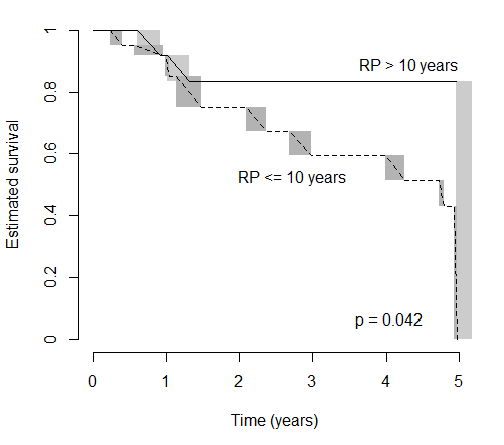


**Supplemental Figure 2 - Survival estimates in relation to the presence of reflux disease at baseline in the validation cohort**


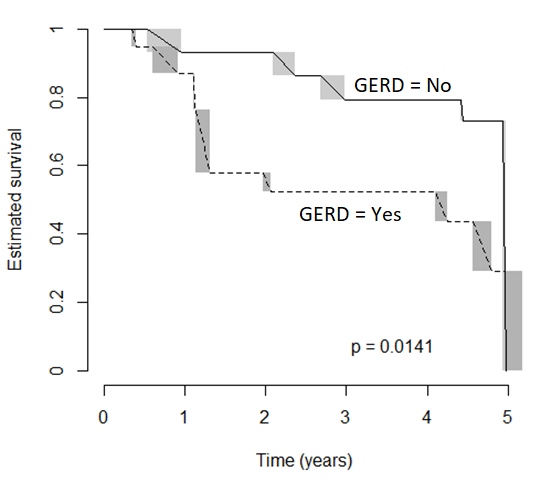

Supplement: Supplementary file 1 — Additional file 1: Supplemental Table 1. Clinical characteristics of patients in the discovery (step 1) cohort. Supplemental Table 2. Clinical variables associated with disease progression in the validation cohort. Supplemental Figure 1. Survival estimates in relation to the duration of Raynaund’s phenomenon at baseline in the validation cohort. Supplemental Figure 2. Survival estimates in relation to the presence of reflux disease at baseline in the validation cohort. [file 13075_2023_2989_MOESM1_ESM.docx]
